# An Immersive and Interactive Platform for Cognitive Assessment and Rehabilitation (bWell): Design and Iterative Development Process

**DOI:** 10.2196/26629

**Published:** 2021-11-03

**Authors:** Vincent Gagnon Shaigetz, Catherine Proulx, Anne Cabral, Nusrat Choudhury, Mark Hewko, Elicia Kohlenberg, Melanie Segado, Michael S D Smith, Patricia Debergue

**Affiliations:** 1 Simulation and Digital Health Medical Devices Research Centre National Research Council Canada Boucherville, QC Canada; 2 Simulation and Digital Health Medical Devices Research Centre National Research Council Canada Winnipeg, MB Canada

**Keywords:** virtual reality, clinical psychology, cognitive assessment, neuropsychology, mental health, cognitive rehabilitation, digital therapeutics, mobile phone, cognitive training

## Abstract

**Background:**

Immersive technologies like virtual reality can enable clinical care that meaningfully aligns with real-world deficits in cognitive functioning. However, options in immersive 3D environments are limited, partly because of the unique challenges presented by the development of a clinical care platform. These challenges include selecting clinically relevant features, enabling tasks that capture the full breadth of deficits, ensuring longevity in a rapidly changing technology landscape, and performing the extensive technical and clinical validation required for digital interventions. Complicating development, is the need to integrate recommendations from domain experts at all stages.

**Objective:**

The Cognitive Health Technologies team at the National Research Council Canada aims to overcome these challenges with an iterative process for the development of bWell, a cognitive care platform providing multisensory cognitive tasks for adoption by treatment providers.

**Methods:**

The team harnessed the affordances of immersive technologies while taking an interdisciplinary research and developmental approach, obtaining active input from domain experts with iterative deliveries of the platform. The process made use of technology readiness levels, agile software development, and human-centered design to advance four main activities: identification of basic requirements and key differentiators, prototype design and foundational research to implement components, testing and validation in lab settings, and recruitment of external clinical partners.

**Results:**

bWell was implemented according to the findings from the design process. The main features of bWell include multimodal (fully, semi, or nonimmersive) and multiplatform (extended reality, mobile, and PC) implementation, configurable exercises that pair standardized assessment with adaptive and gamified variants for therapy, a therapist-facing user interface for task administration and dosing, and automated activity data logging. bWell has been designed to serve as a broadly applicable toolkit, targeting general aspects of cognition that are commonly impacted across many disorders, rather than focusing on 1 disorder or a specific cognitive domain. It comprises 8 exercises targeting different domains: states of attention (Egg), visual working memory (Theater), relaxation (Tent), inhibition and cognitive control (Mole), multitasking (Lab), self-regulation (Butterfly), sustained attention (Stroll), and visual search (Cloud). The prototype was tested and validated with healthy adults in a laboratory environment. In addition, a cognitive care network (5 sites across Canada and 1 in Japan) was established, enabling access to domain expertise and providing iterative input throughout the development process.

**Conclusions:**

Implementing an interdisciplinary and iterative approach considering technology maturity brought important considerations for the development of bWell. Altogether, this harnesses the affordances of immersive technology and design for a broad range of applications, and for use in both cognitive assessment and rehabilitation. The technology has attained a maturity level of prototype implementation with preliminary validation carried out in laboratory settings, with next steps to perform the validation required for its eventual adoption as a clinical tool.

## Introduction

### Background

Mental health issues are increasing worldwide [1]. In middle- and high-income countries, 50% of the general population will experience at least one mental health disorder during their lives [2]. Mental illness impacts national productivity, estimated to be up to a 4% loss as measured by gross domestic product [3]. The cumulative economic output loss associated with mental disorders between 2011 and 2030 is projected to be US $16.3 trillion worldwide, putting it in close contest with cardiovascular disease, which is the leading health care burden [2]. Mental health disorders encompass many conditions, challenging people throughout their lives by impacting their ability to learn, build flourishing lives, and age gracefully into their senior years [4]. Although mental health issues are prevalent, they remain difficult to assess and treat.

To best manage a mental health condition, it is important to understand the different ways in which functioning is impacted in an individual. A core feature of psychopathology is cognitive dysfunction, in which impairments can occur broadly and nonspecifically among domains such as attention, response inhibition, and visual memory, cutting across disorder boundaries [5]. Current assessments of cognitive functions, traditionally including in-person clinical evaluations consisting of a battery of pen-and-paper and 2D computerized cognitive tasks [6,7], may not be able to capture the complex processes underlying behavior because they are based on a common set of neuropsychological tools evaluating unitary cognitive constructs [8]. On the other hand, looking at treatment, it becomes evident that cognitive rehabilitation has emerged as a promising strategy. It is based on the premise that repeated practice of tasks targeting deficits can lead to improvements in specific cognitive domains [9]. However, it remains unclear whether these improvements translate into real-world functioning.

Neuropsychological assessments are currently experiencing a shift, moving away from traditional *construct-driven* pen-and-paper paradigms toward tests that are representative of everyday life, attracting the use of immersive, otherwise known as extended reality (XR) technologies [10-12]. In particular, virtual reality (VR) allows for almost complete sensory immersion with vast design possibilities and tight experimental control, making it ideal for assessing cognitive functioning in the performance of simulated real-life tasks. The ability of VR to deliver and control stimuli while capturing responses with high fidelity during an exercise, provides a controlled and repeatable tool that is unavailable in traditional testing methods. The opportunities VR provides for both assessment and rehabilitation have led to growing interest from the research and clinical neuropsychology communities in recent years [13]. The feasibility of using VR for cognitive assessment and care has been demonstrated across various cognitive domains, such as attention [6,14,15], executive functions [16], memory [11,17,18], and spatial abilities [19]. Moreover, in an extensive review describing the status of clinical VR, Rizzo et al indicate that studies on the use of VR for cognitive assessment have demonstrated construct validity and discrimination of clinical groups from healthy controls, while VR cognitive rehabilitation studies, though promising, have produced mixed results [20]. In a systematic review of VR cognitive rehabilitation, Larson et al [21] identify a few randomized controlled trials demonstrating effective training for memory [22-24], executive functioning [25], and visual attention [26] and conclude that further studies in the field are needed.

Although existing solutions have shown great promise for the use of digital cognitive health interventions in general, and even VR interventions specifically, these interventions have not yet been widely adopted. The majority of the VR platforms mentioned above comprise a single exercise tailored to a specific disorder, require manual exercise reconfiguration to support repeated measures, and support limited and often specific user display and interaction hardware [6,21,27,28]. Here, we outline a co-design development framework for a cognitive care platform and the platform developed using this process that addresses these limitations. The development framework includes an interdisciplinary team to match clinical intentions with exercise software design for stimuli and measurements, and a set of clinical partners to configure and validate multipurpose exercises for specific use cases.

### Objective

The aim of this work is to create a toolkit for clinicians to perform assessment and rehabilitation on a platform enabled with immersive technologies. Because of the novel, interdisciplinary nature of developing software for immersive cognitive care, a secondary goal is to outline a process for the iterative development of cognitive care software in collaboration with domain experts.

## Methods

**Figure 1:**
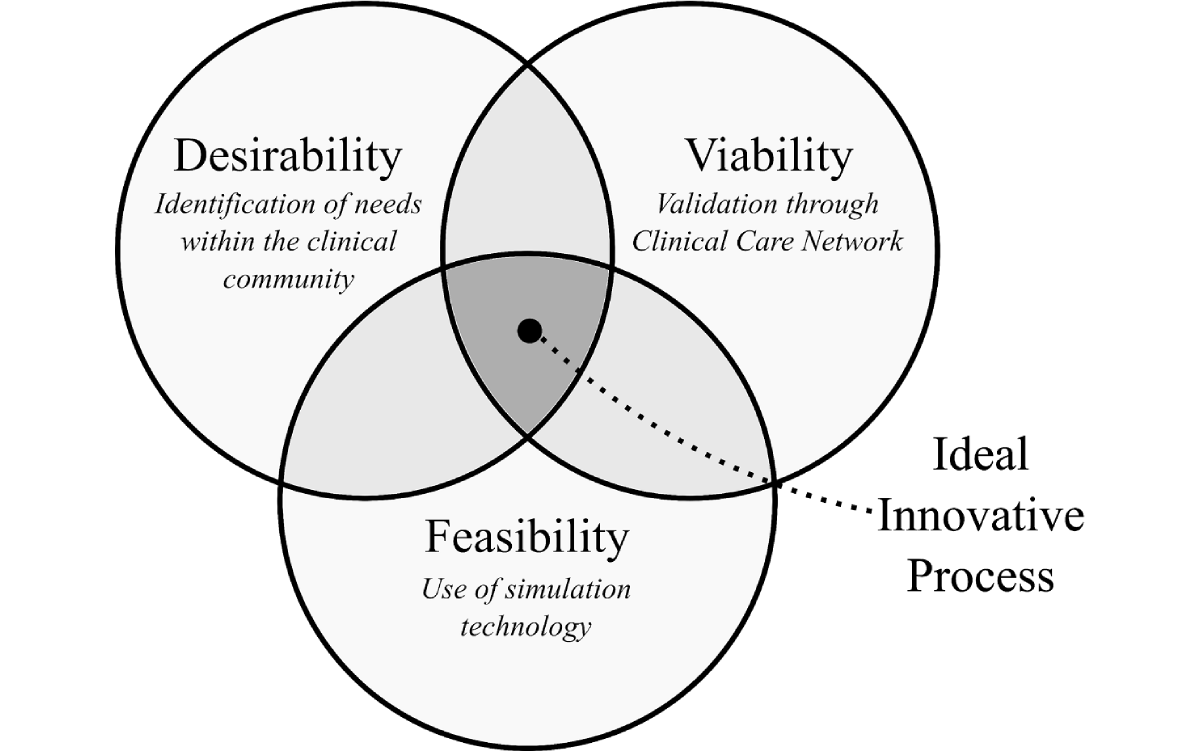
Venn diagram showing innovative solution sweet spot that lies at the intersection of desirability, viability, and feasibility.

A human-centered approach and design thinking were used to identify key requirements for the proposed platform such that it would satisfy the criteria of being desirable, viable, and feasible (Figure 1). When these components are balanced, one can arrive at an innovation process that integrates the needs of the end users as well as the potential of technology in a sustainable fashion [29].

**Figure 2:**
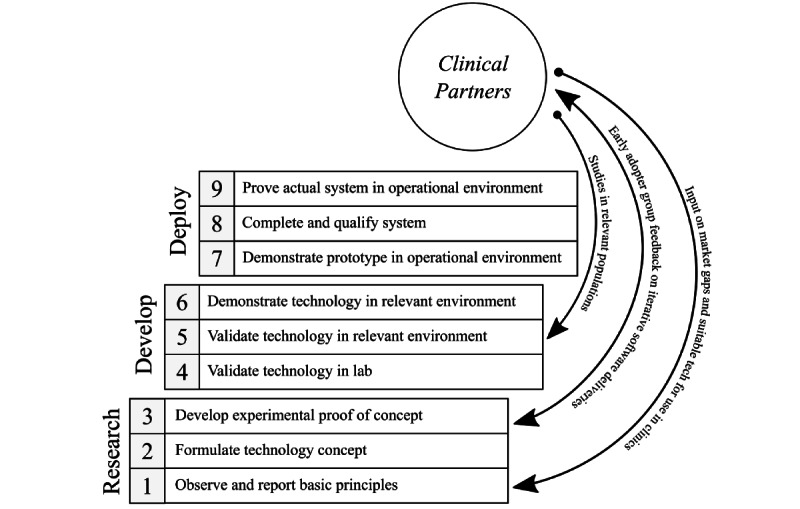
Schematic demonstrating the stages of clinical collaboration at the different TRL: early TRL (1-3: Foundational research), mid TRL (4-6: Technology development) and late TRL (7-9: deployment). TLR: technology readiness levels.

Specifically, an interdisciplinary approach [30,31] previously employed by the National Research Council Canada (NRC) was leveraged to develop the bWell cognitive care platform. This approach has been successfully applied by the NRC to create surgical simulation platforms [30-35], most notably for neurosurgery, NeuroTouch (now distributed worldwide as NeuroVR by CAE Healthcare). For a feasible solution, the technology was built on the team’s strengths in simulation. Over the years, the team has developed skills and expertise in assembling the pieces required for user interactivity in realistic real-time simulation, engaging users through multiple senses (visual, audio, and touch). Desirability, or the need for the platform within the clinical community, was determined through active discussion with clinical care providers and by identifying gaps in existing solutions in the market. In the interest of viability, early on, the team established long-term collaborative research agreements with 5 world-renowned clinical sites across Canada and 1 in Japan—a cognitive care network (CCN). The CCN consists of an early adopter group that has provided domain-specific perspectives as well as feedback on iterative deliveries of the platform as bWell development progressed. The CCN is currently launching several studies investigating the content of bWell exercises, as well as its use, specific to target populations. In addition, the technology readiness level (TRL) framework [36] has been used to integrate and structure the stages of clinical collaboration within the technology development phases according to the level of maturity (Figure 2).

## Results

### Technology Readiness

The development of the bWell platform began in 2017, and activities to date have focused on research to demonstrate technical feasibility as defined in the early TRLs. During this phase of technology development, 4 main activities were advanced: (1) identification of basic requirements and key differentiating criteria (TRL-1), (2) prototype design and foundational research to implement key components (TRL 2-3), (3) testing and validation in laboratory settings (TRL 3-4), and (4) the establishment of the CCN early adopter group. The CCN was formed in 2019 to prepare for the time when the platform would reach an intermediate TRL (TRL 4-6), ready to be shifted from validation in the laboratory to validation in clinical settings.

### Identification of Requirements and Differentiators

The fundamental objective of this work is to harness the affordances of immersive technologies to enable assessment and therapy that can meaningfully align with real-world problems in cognitive functioning. By creating simulations that engage users through multiple senses (visual, audio, and touch) while permitting natural movement, immersive VR creates a sense of presence (*being there*) that elicits a genuine response in an individual [37]. The use of VR environments also permits tight experimental control, which makes it feasible to measure and study everyday functioning that can otherwise be prohibitively difficult in real-world settings.

Taking into consideration the needs for cognitive care, technology affordances and innovation potential, four main requirements were identified for the bWell platform:

#### Requirement 1: Support for Multiple Hardware Platforms and Different Modes of Immersion (Fully, Semi-, or Nonimmersive)

Support for third-party hardware with varying levels of technical maturity facilitates the planning of clinical interventions and research in the ever-changing technical landscape of VR hardware. In addition, different modes of immersion allow for flexible content delivery in cases where immersive technology is not available or where a fully immersive environment is not well tolerated by a patient. Integrating this support also enabled the use of a range of low-cost to high-end consumer devices for clinical and home settings.

#### Requirement 2: A Suite of Customizable Tasks

This approach allows clinical partners to choose from the available tasks and options based on the needs of a specific patient or target clinical population (eg, pediatric and older adult patients). Common core features required by all tasks were standardized to enable a faster, more agile software development process and to open up possibilities for adding customized features. Tasks were selected to address aspects of cognition common to a variety of mental health disorders rather than a specific disorder. Additionally, configurable exercises permit pairing standardized assessment with corresponding adaptive and gamified variants for therapy, providing therapeutics that do not stand in isolation from assessment.

#### Requirement 3: A Clinician-Facing UI to Control the Task Parameters

This interface had to contain a wide range of adjustable parameters, exposing the extensive design options afforded by the platform. Exposed parameters would permit the clinician to administer and prescribe interventions (dosing, duration, and frequency) as well as facilitate their surveillance and control for a trial.

#### Requirement 4: Data Logging Mechanism

Behavioral and experimental data recorded with precise timing are required for the study of cognitive processes. To enable intra and intersubject analysis of user response and physical interaction, recording of movement data, user performance, and simulation cues and events were also required.

### Implementation

The implementation consisted of developing a platform enabled by immersive technologies and translating the requirements identified through the active co-design process (outlined above) into hardware and software components, including the design of the content.

**Figure 3:**
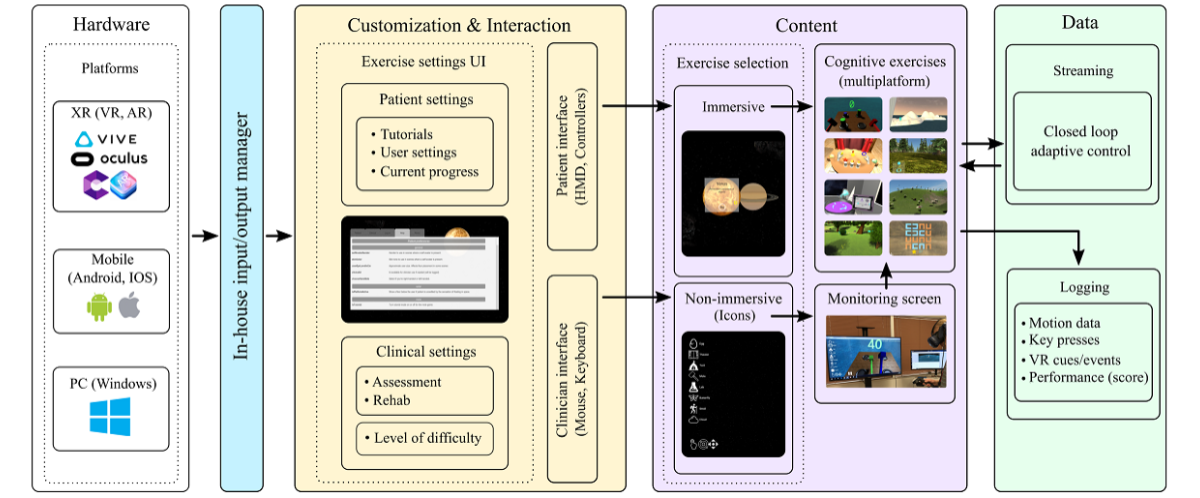
bWell architecture components (Unity 3D engine): (1) in-house input or output manager for hardware, (2) comprehensive user interface for customization of patient and clinical settings, (3) content displayed immersively to the patient or nonimmersively for clinician administration, control and monitoring, and (4) resulting data are streamed for real-time adaptive control and automatically logged for offline analysis. AR: augmented reality; HMD head-mounted display; VR: virtual reality; XR: extended reality.

bWell was developed using the Unity 3D game engine and was implemented with multiple components (Figure 3). Tasks were developed around a generic core that defines the flow and interactions between different software and hardware components. To support a variety of hardware (XR headset, mobile device, PC), an in-house input manager was developed. This component is an abstract layer that maps device inputs and outputs to a functionality in the software. The clinician and the patient interact with the platform through different interfaces to facilitate patient-clinician interactions. When the platform is launched, the clinician can access a nonimmersive user interface (UI) to configure the trial settings required to customize the intervention for the patient. The patient can interact within the virtual environment for the selection of exercises using immersive hardware. The content component contains the exercises and the monitoring system. The exercises include the virtual environment, task logic (rules and goals), instructions, and exercise-specific interactions. When using an immersive headset, the patient’s point of view in their head-mounted display (HMD) is also displayed on a second screen to enable monitoring. This is the same screen through which the clinician can access the exercise settings. In addition, the clinician can launch a task by clicking on the representative 2D icon in the overlay. The final component concerns the data. Streaming data are used in a closed loop to control the evolution of the exercises based on user performance. Detailed data including motion, key presses, cues, events, and patient performance are also logged into files for post analysis.

### Development Process

The research team made use of agile software development, including feature-oriented code implementation, common code repository, software testing, user tests, bug tracking, and frequent software releases. Standardized user testing was developed to solidify the integration of hardware and software features. Individuals new to the platform were included as testers to reinforce usability and to reveal issues that those familiar with the system could no longer identify. Furthermore, to help maintain the major features of each of the exercises and the core features, unit tests developed with the integrated Unity 3D tools were put in place to automate the process. To obtain active input from domain experts, the agile methodology included regular delivery of technology to early adopters to obtain iterative feedback. This feedback informed the successive phases of software development.

### Prototype Development

**Figure 4:**
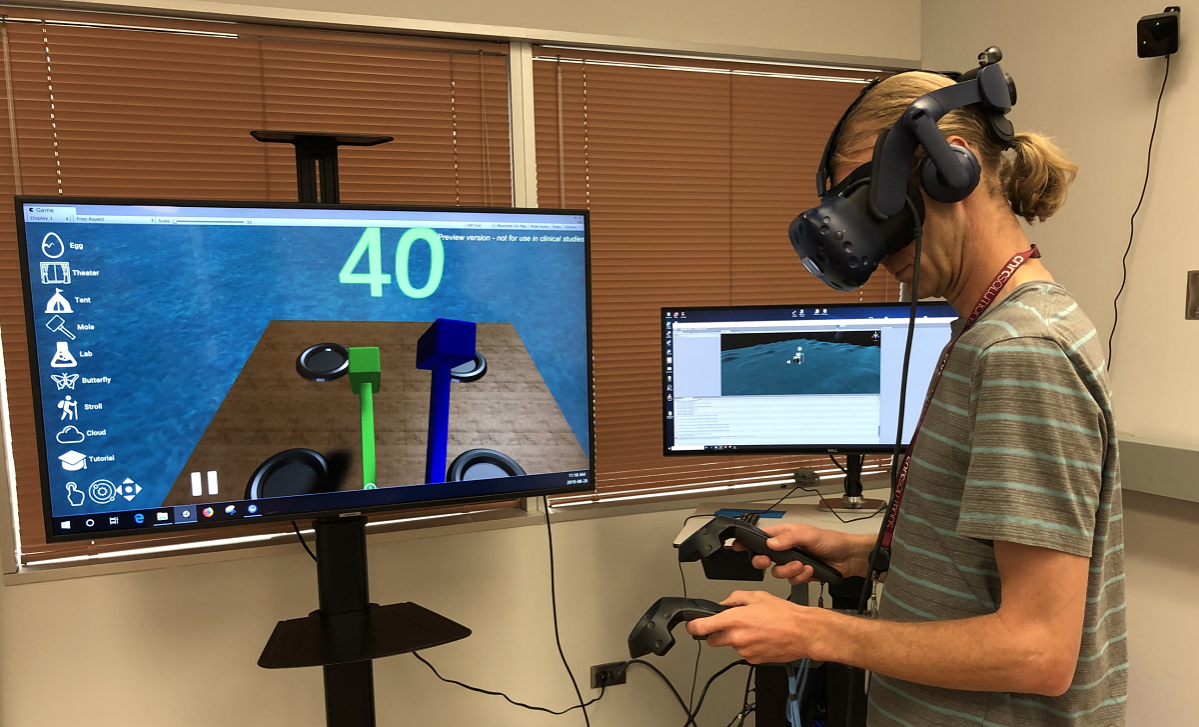
bWell laboratory set-up with a user immersed in a virtual reality environment and the point of view displayed on an auxiliary monitoring screen.

The technical feasibility activities for bWell led to the development of a prototype at a TRL-4 maturity level that has been tested and validated in a laboratory environment (Figure 4).

The prototype was designed to be administered by clinicians in various clinical environments. Its architecture was kept flexible, accommodating various input modalities and administration hardware, because it was identified that each clinic had different hardware needs depending on their patient constraints or limitations, price, availability, and the hardware that they already owned. Thus, the prototype was implemented as multimodal (fully, semi-, or nonimmersive) and multiplatform (Oculus, HTC Vive, Hololens, tablet [iOS and Android], and desktop). Automatic detection of the connected devices is executed when the platform is launched, making it a seamless feature for the user, thereby facilitating the long-term goal of functioning in a home environment with remote monitoring by a clinician.

### Exercises

**Figure 5:**
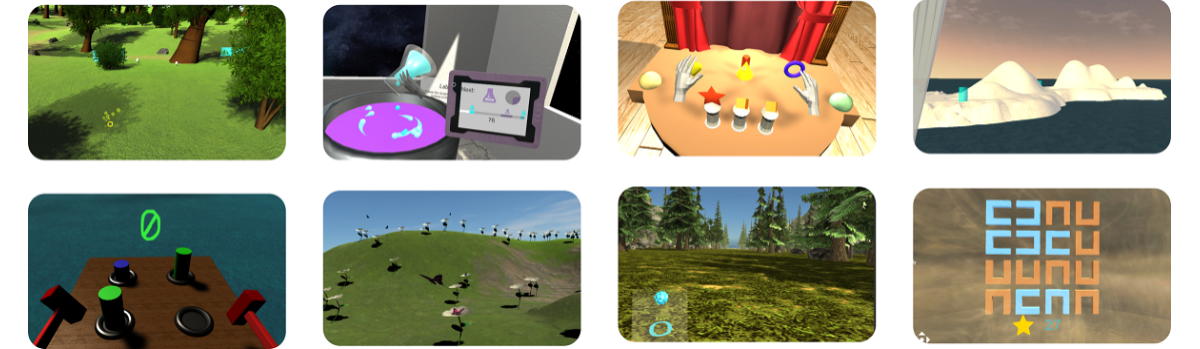
Cognitive exercises: (Top row) states of attention (Egg), multitasking (Lab), visual working memory (Theater), relaxation (Tent); (Bottom row) response inhibition and cognitive control (Mole), self-regulation (Butterfly), sustained attention (Stroll), visual search (Cloud).

The exercises provided in the prototype were developed to target cognitive domains common to multiple mental health disorders, including attention, memory, and executive control. To promote presence and immersion in the simulation, all exercises make use of multisensory feedback (visual, audio, and touch). Some exercises were designed to target a specific domain of interest, whereas others simultaneously engage multiple cognitive domains to be representative of everyday tasks. A total of 8 exercises were implemented (Figure 5).

#### States of Attention (Egg)

The user must first scan the environment for eggs. They are then required to direct and hold their gaze on a located egg long enough to make it hatch (attentional focus). Audio and visual distractors in the environment challenge the user, and bonus points are awarded if the user reacts to a cue while fixating (covert attention).

#### Multitasking (Lab)

The user must complete 2 recipes simultaneously to investigate their ability to accomplish a range of tasks by swapping between them strategically or by planning the order in which they can be performed most efficiently. This requires the user to closely follow the recipe steps (ability to monitor) displayed on the virtual tablet screens in front of them.

#### Visual Working Memory (Theater)

Inspired by matching tasks for visual and short-term memory, the user is presented with target shapes ordered from left to right. After a set viewing time, the targets are hidden. After a specific time (delay recall), objects fall into view of the user, some of which are the target and others are not (comparison objects). The user is required to select the targets from all the objects and place them in the order presented within a limited time.

#### Response Inhibition & Cognitive Control (Mole)

A Whack-A-Mole variation is used where the user has a hammer in each hand and has to hit cylinders that pop up in front of them. The colors of the hammers change over time, and the cylinders also have different colors. The user should only hit cylinders with a hammer of the same color (go signal).

#### Self-regulation (Butterfly)

The user engages in motor self-regulation through an activity that rewards self-restraint. The user is instructed to catch butterflies with a net but must do so in a gentle fashion because brisk movements scare them away. A motion speed indicator is visible on the net to promote self-awareness. The user can monitor, control, and inhibit unproductive motor responses that may be triggered when the butterflies are near.

#### Sustained Attention (Stroll)

This is an immersive version of a sustained attention to response task, a go or no-go task with infrequent no-go events to measure user attention. The user is provided a self-avatar, taking a stroll in a natural scene. Shapes continuously appear in front of the user, and a button must be pressed when each new shape appears, except when it is a green diamond.

#### Visual Search & Attention (Cloud)

This is an immersive version of a visual search and attention test, in which a grid of orange and blue *U* shapes is displayed in front of the user. The user must find the only blue *U* shape that points upward or downward and respond with the corresponding up or down response on their controller. Orange shapes and blue shapes pointing left or right are distractors that must be ignored.

#### Relaxation (Tent)

In relaxation and sensory exploration, the user is immersed in scenic views and asked to look around while focusing on their breathing. A rhythmic object is present to guide their breathing pace. Close adherence to the rhythmic object should have a calming effect. The user is free to explore the scene in which they are or to select a different scene from a set of options presented to them as pages in a book. This exercise was also designed for eventual self-guided stress management, where future efforts will include the integration of heart rate variability biofeedback based on slow-paced breathing within virtual nature scenes.

### Clinician Customization and Monitoring Interfaces

**Figure 6:**
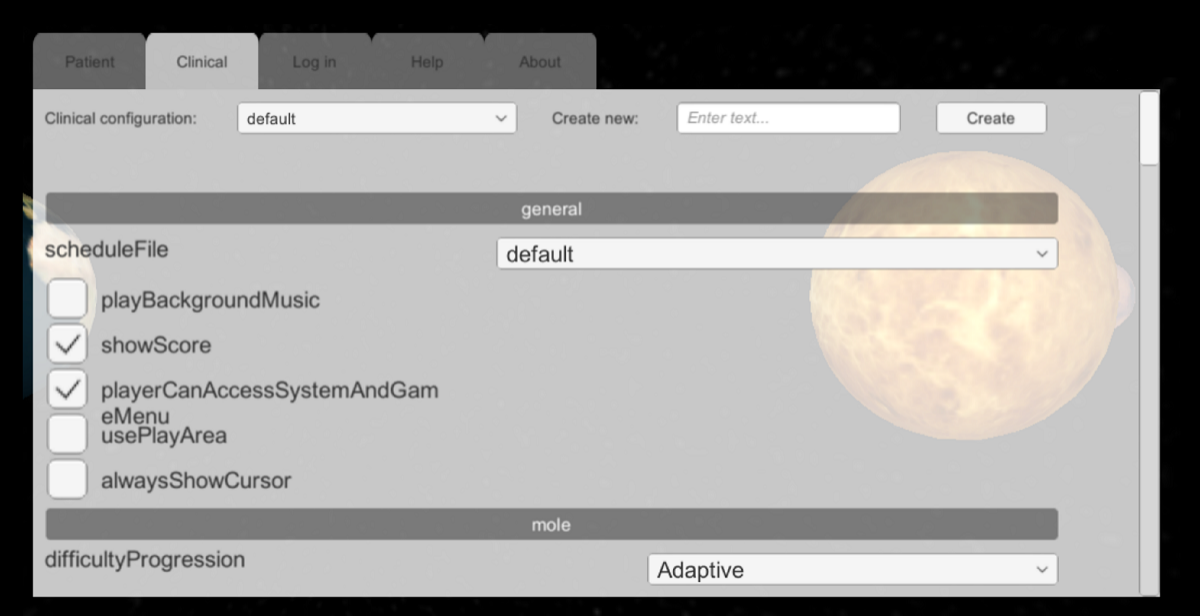
Clinician-facing user interface with clinical exercise settings interface under Clinical tab.

**Figure 7:**
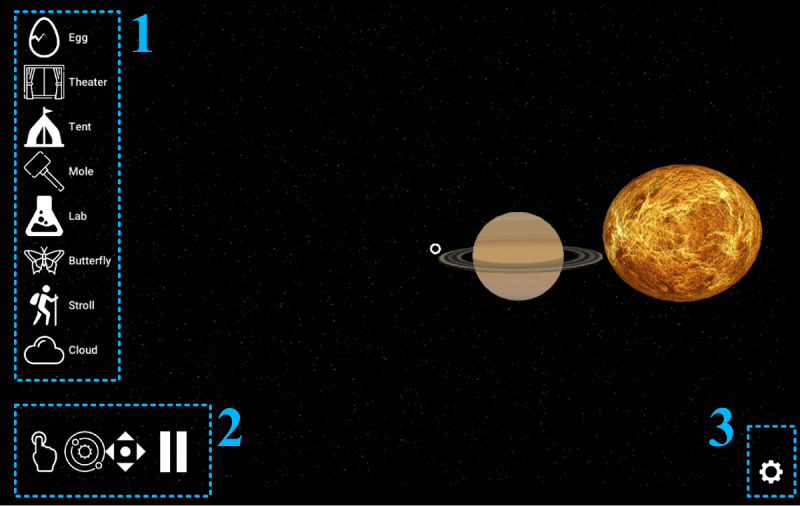
Clinician user interface 2D icons: (1) exercise selection, (2) real-time intervention, and (3) settings user interface menu access.

The clinician-facing UI was developed to allow the exercise parameters to be customized and for functionalities to be turned on or off during the exercises. A scrolling menu (Figure 6) provides access to the settings to design the variants of a given exercise, or establish the dosing of an intervention, pairing standardized assessment with corresponding adaptive and gamified variants for personalized rehabilitation training. The settings can be saved and shared, allowing multiple clinical sites to conduct studies with standardized configurations. While a patient participates in a bWell trial with an HMD, a white overlay of clickable icons are available to the clinician to assist with operating and controlling the VR experience received (Figure 7). The icons allow the clinician to launch a specific exercise (left panel) and to intervene if needed, such as start or end the exercise, recenter the user in the immersive environment, or pause the session (bottom panel).

### Task Parameters

bWell was designed to serve as a toolkit; so, task parameters were designed to be highly configurable. Several personalization elements were included to promote a sense of embodiment. The self-avatar can be personalized with elements such as gender, skin color, height, and dominant hand that can be modified in a patient settings tab added to the clinician-facing UI. bWell’s design also considers potential physical limitations of users, giving them the option to play while standing or sitting. The patient settings can also be used to adjust the height of certain elements inside an exercise if the auto-adjusting features are insufficient. In addition, the patient VR interactions in bWell use multiple input types, such as gaze direction, teleportation, and grabbing or hitting objects, to accommodate participants with conditions that limit dexterous hand movements.

Task difficulty parameters were configured to allow for personalized assessments and rehabilitation plans. Difficulty levels can be set at fixed levels by the clinician, ensuring trial reproducibility for comparison with past performance or across participants, or set to use an adaptive algorithm configured to achieve an 80% success rate. Intra and intersession difficulty progression settings are also available.

All task parameters are logged using a completely automated logging system to facilitate offline analysis. In addition to logging exercise settings, event orders, and difficulty levels, the system records an array of timestamped, synchronized data, including motion tracking, exercise events, cues presented to the user, and performance measures. The logs, anonymized for confidentiality, are output as JSON and CSV files, which are compatible with standard data analysis software.

Other features were included to add variety to gameplay and to promote the repetition of tasks typically required in cognitive rehabilitation protocols. Gamification elements, such as rewards and animations, were integrated to promote adherence and engagement and to provide feedback on performance. Visual cues, audio cues, and sometimes distracting elements were also incorporated. In certain exercises, the displayed virtual environment can be explored with a teleportation feature, or the user can select between different virtual environments.

### Intervention Modes

bWell is designed to operate in three modes: (1) tutorial, (2) assessment, and (3) rehabilitation. Each mode is designed to address a specific set of clinical requirements determined through early adopter feedback.

#### Tutorial Mode

**Figure 8:**
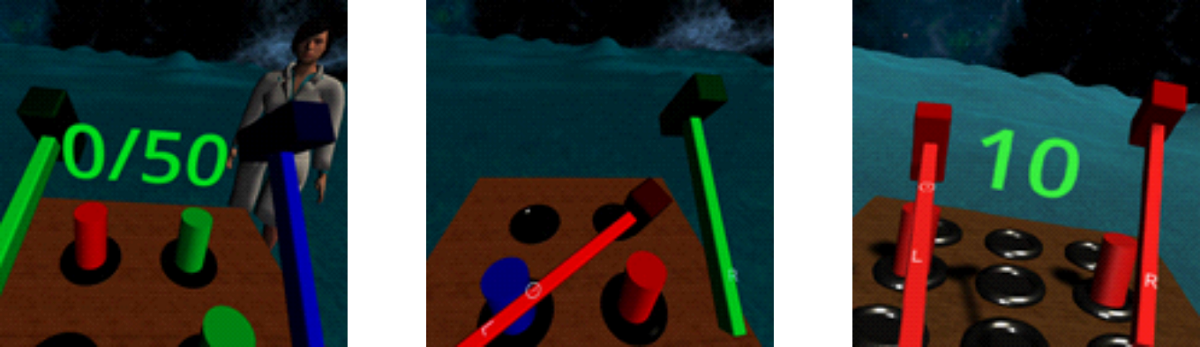
bWell Mole exercise as an example of modes and configurable parameters. The left image shows the tutorial mode with the score that must be achieved before the exercise can begin. The humanoid avatar provides instructions and verbal feedback on successes and failures. The center image shows the mole exercise in progress at a fixed, low difficulty level (4 cylinders) without any feedback on successes and failures. The right image shows the mole exercise in progress with adaptive difficulty leveling and a score visually displayed. This configuration aims for gamified rehabilitation.

In the tutorial mode, participants engaged in structured learning of the required actions for each task. This was implemented to ensure that participants began assessment and rehabilitation exercises with comparable levels of familiarity with the actions required to complete the exercises, even if they had different baseline levels of familiarity with XR. During the tutorial for each exercise, a standardized set of instructions is presented in writing and verbally by a humanoid avatar. If an action was not executed after a specified time interval, a revised version of the instructions was presented to increase the likelihood that participants properly understood what was required. The humanoid avatar also provided verbal feedback about successes and failures throughout the tutorial (Figure 8, left). The only way to progress through the tutorial is by successfully executing the specific required actions, at which point the participant can continue engaging in free practice without any specified goal. The clinician can then transition from the tutorial mode to the assessment or rehabilitation mode through the clinician-facing UI.

#### Assessment Mode

In the assessment mode, participants completed the exercise with events always occurring in the same order and with the same timing (ie, based on a fixed randomization seed) and with fixed difficulty levels [38] (Figure 8, center). The seed number that determines the order and timing of events can be modified by the clinician such that assessments can be pseudorandomized across sessions or participants. The clinician configures the settings for each difficulty level to best meet their assessment needs. Because the presentation to each user is consistent, it is possible to assess user performance against established results or in comparison to others in the same study. For example, commission errors (incorrect hits) are of particular interest when investigating cognitive control.

#### Rehabilitation Mode

In the rehabilitation mode, the emphasis is on parameters that promote patient adherence by increasing engagement and providing feedback on performance. Adaptive algorithms adjust current level of difficulty based on performance to ensure that it is never too easy (boring) or too hard (demoralizing) for the user [39]. For enhanced motivation, real-time feedback is provided with level or score changes as well as success and error indications (Figure 8, right). Finally, in the rehabilitation mode, the series of events in each session is randomized to avoid redundancy. Therefore, even if the progress is reset between sessions, a patient should never see the exact same series of events twice.

### Acceptability Study Results

The authors conducted 2 preliminary acceptability studies with healthy participants and have described them in previous reports [40,41]. These studies showed that using immersive VR for clinical applications is not only technically feasible but also well tolerated and has advantages over traditional 2D equivalents. The first study showed that subjective reports of engagement when performing a task in bWell (an immersive environment) were greater than those when performing the same task on a tablet (a nonimmersive environment) [40]. The second study showed that two types of passive displacement, linear and sinusoidal walking vection, did not increase subjective reports of cybersickness during a visual attention task [41].

The results of these preliminary studies demonstrate the acceptability of the bWell platform. The careful attention taken during the design regarding cybersickness has shown successful administration even in participants who reported being highly susceptible to motion sickness. bWell tasks in immersive VR, both in static scenes and those involving more complex user motion, were well tolerated and engaging for healthy participants and provided the required support for testing in clinical populations as the next steps. In addition, as it was determined that engagement is not the same for immersive versus nonimmersive delivery of the exercises, the fully semi-, or nonimmersive modes may result in different user performances. As such, and in particular for cognitive assessment, comparisons of task results should be performed within a given mode of immersion.

Although bWell has shown general acceptability and tolerability, a small number of users experienced mild cybersickness. The Biomedical Data Intelligence team at the NRC investigated the use of an avatar within the bWell environment to monitor user discomfort. The dialog agent provides instantaneous and interactive assistance to users in the form of personalized advice on symptom relief [42]. The results show promise for the development of virtual agents for cognitive self-care and will be further explored for use within bWell.

### Cognitive Care Network

**Figure 9:**
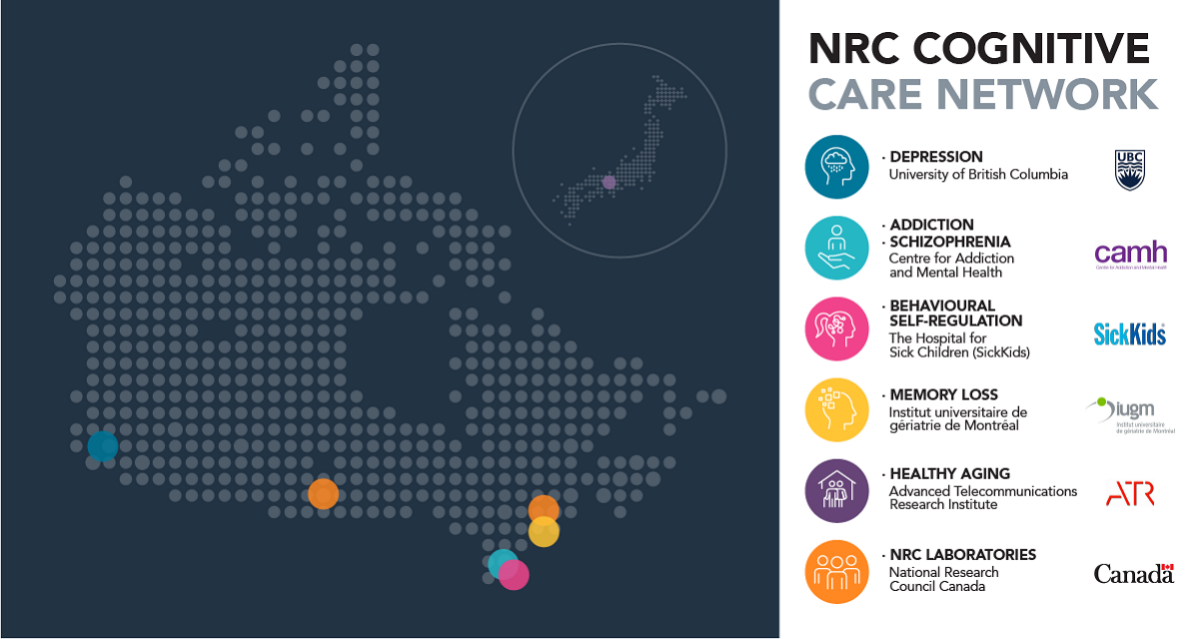
Cognitive Care Network (CCN) sites. The CCN includes 5 clinical partners at 4 sites across Canada (CAMH, IUGM, SickKids, and UBC) and 1 site in Japan (ATR). Sites cover a broad range of expertise and are critical for the iterative development of bWell.

The prototype is currently being taken beyond the laboratory and into clinical settings. To provide domain-specific expertise and clinical validation, a CCN was assembled consisting of 4 institutions across Canada and 1 in Japan (Figure 9). They have expertise in addiction, schizophrenia, memory and mild cognitive impairment in older adults, executive functioning in pediatric populations and major depressive disorders with backgrounds in neuropsychology, psychiatry, and clinical psychology.

Evaluation of bWell by clinicians began in 2019 to provide domain-specific input throughout the bWell development process. bWell has thus far been installed at 4 locations, and ongoing feedback from early testing at these installations has been incorporated in iterative improvements. Adaptations of bWell exercises to target clinical populations have begun. These activities are structured according to the Birckhead et al VR trial methodology [43], including co-design of content with patients and early studies (feasibility, acceptability, tolerability, and initial clinical efficacy), as well as randomized controlled trials investigating the outcomes of the use of bWell. As these activities are currently underway, they are beyond the scope of this paper.

## Discussion

### Principal Findings

The use of digital solutions has the potential to address the current gap in mental health care resources. To this end, digital therapeutics have entered the pharmaceutical landscape [44]. Web-based and mobile apps can improve the accessibility and affordability of care and can help keep patients motivated and engaged during interventions. To maximize the latter value, digital solutions have taken creative approaches, using game-based therapy or leveraging technology to create more immersive experiences [45]. XR platforms on the market currently include mental health care for behavioral health (BehaVR), autism and developmental delay screening (Cognoa), and stress management (Healium). Although XR solutions are currently in the stages of demonstrating evidence on the benefits of use, some mobile-based digital therapeutics have reached regulatory approval [46]. For example, Pear Therapeutics has received authorization from the US Food and Drug Administration (FDA) for prescription-based digital therapeutics for substance abuse disorders and chronic insomnia. Moreover, in 2020 the FDA permitted the marketing of the first game-based digital therapeutic by Akili Interactive to improve attention function in children with attention-deficit/hyperactivity disorder [47,48].

A primary driver for the use of VR technology is its capacity for sensory immersion with tight experimental control, making it feasible to test or study cognitive and sensory-motor functioning that is typically prohibitive in real world settings because of the unpredictable and uncontrolled events that occur in everyday life. Immersive and engaging tasks were selected in bWell to encourage meaningful user interactions. The simulated scenes were also designed to be multisensorial and interactive to permit naturalistic movement, increasing the likelihood that skills learned within VR would be transferable to real life.

Another driver for the use of simulation technologies is the ability to capture rich behavioral data simultaneously. As users interact in virtual environments, all activities can be recorded for analysis. bWell was implemented with two data workflows—one with data streaming for real-time adaptive exercises and the other with data logging for offline analysis. In bWell, streaming data are used in a closed loop to adapt the exercises in real time, targeting patient-specific rehabilitation. Currently, data on user performance are used as input for adaptive algorithms that adjust the difficulty levels of the exercise accordingly. The integration of wearable, physiological sensors in XR scenes is also currently in progress to enable biofeedback. In this case, features derived in real time from sensor data are used as input to adapt the exercises. As part of the collaboration with the Advanced Telecommunications Research Institute from the CCN, new exercises are being developed for cognitive training using an electroencephalography-based brain-machine interface to control a virtual third arm [49]. This work has broad applicability as it provides a novel form of multitasking training for elderly users to cognitive load training in operational environments.

In bWell, data are also currently being logged for offline analysis, with information including motion, key presses, VR cues, events, and patient performance. In this workflow, the goal is to derive features from sensor data that cannot be obtained from real-time processing. For example, classification of user states can be obtained with predictive models built using techniques performed offline, such as cognitive modeling and machine learning. With user state obtained from quantitative measures, such as user reaction to an increase in exercise difficulty or adverse response, VR assessments coupled with physiological sensors can provide more objective measures of individual function than a traditional self-report, which is known to be an unreliable index of functional outcomes [50]. By collecting behavioral and physiological responses during carefully designed VR interventions, the long-term vision is to create closed-loop adaptive digital interventions for characterizing and treating cognitive deficits, as well as enabling treatment providers to predict real-world clinical outcomes.

During the development of the platform, two lessons were learned. First, design with a generic core and providing users with customizable options is a valuable approach for developing applications for a variety of treatment providers. To accomplish this, the bWell platform was designed to target specific cognitive domains rather than specific pathologies. Clinicians select exercises from the available ones to address the needs of a particular patient. This choice provides an opportunity to address symptoms rather than disorders. The treatment provider can further customize individual exercises by choosing from the configurable settings, such as enabling 1 of the 3 basic modes (tutorial, assessment, and rehabilitation) or turning specific functionalities on or off. In the study of cognitive functioning, this means that one is able to design a paradigm specific to a research question by choosing the virtual environment (eg, game-based or real-world setting), the type of experimental stimuli (eg, target, distractors, cueing, and feedback), the time and type of presentation (multisensorial or not), and the type of user response (eg, aim to minimize errors or perform as quickly as possible).

Second, avoiding dependence on specific hardware is advantageous when developing with XR. The implementation in bWell was hardware agnostic, multimodal, and multiplatform, accommodating the range of specifications coming from different potential clinical applications and increasing its staying power in the rapidly changing technological landscape. The work done on bWell focused on the use case where clinicians administer the intervention. However, the hardware agnostic core of the platform considers its eventual use at home with remote monitoring by clinicians. bWell’s core was also designed to accommodate the evolution of commercially available HMDs. HMDs have come a long way, from unwieldy, tethered devices to performant and comfortable standalone units aimed at the mass market. bWell’s core also allows for other modes of content delivery, such as AR or mobile, when immersive VR is not suitable. For instance, Google AR glasses have shown to be promising for teaching children on the autism spectrum to recognize emotions in real time [51]. Given that a headset obscures facial expressions, the use of VR is not suitable in this context. The use of bWell as a mobile app might also facilitate pervasive use at home, given the ubiquity of smartphones. Nonimmersive XR may also provide an option for those susceptible to cybersickness in immersive environments. Thus, further advancement of bWell is planned in these directions.

### Conclusions

bWell, developed by the NRC, is an immersive and interactive cognitive care platform that delivers assessment and therapeutic tasks as a multisensorial experience (visual, auditory, and touch). The technology has attained the maturity level of prototype implementation with preliminary validation carried out in laboratory settings. A CCN of early adopters was formed to evaluate the system and access domain-specific perspectives. Within this network, 4 installations of bWell prototypes have been completed, and the next steps have begun to adapt the system and to co-design content targeting specific clinical populations. In addition, we plan to perform the validation required for the eventual adoption of bWell as a clinical tool.

## Abbreviations

AR: augmented reality
CCN: cognitive care network
FDA: US Food and Drug Administration
HMD: head-mounted display
NRC: National Research Council Canada
TRL: technology readiness level
UI: user interface
VR: virtual reality
XR: extended reality
